# Acceleration of Mechanistic Investigation of Plant Secondary Metabolism Based on Computational Chemistry

**DOI:** 10.3389/fpls.2019.00802

**Published:** 2019-06-26

**Authors:** Hajime Sato, Kazuki Saito, Mami Yamazaki

**Affiliations:** ^1^ Graduate School of Pharmaceutical Sciences, Chiba University, Chiba, Japan; ^2^ Center for Sustainable Resource Science, Advanced Elements Chemistry Laboratory, Cluster for Pioneering Research (CPR), RIKEN, Saitama, Japan; ^3^ RIKEN Center for Sustainable Resource Science, Yokohama, Japan

**Keywords:** biosynthesis, computational chemistry, density functional theory, molecular dynamics simulation, quantum mechanics/molecular mechanics, plant, secondary metabolite

## Abstract

This review describes the application of computational chemistry to plant secondary metabolism, focusing on the biosynthetic mechanisms of terpene/terpenoid, alkaloid, flavonoid, and lignin as representative examples. Through these biosynthetic studies, we exhibit several computational methods, including density functional theory (DFT) calculations, theozyme calculation, docking simulation, molecular dynamics (MD) simulation, and quantum mechanics/molecular mechanics (QM/MM) calculation. This review demonstrates how modern computational chemistry can be employed as an effective tool for revealing biosynthetic mechanisms and the potential of computational chemistry—for example, elucidating how enzymes regulate regio- and stereoselectivity, finding the key catalytic residue of an enzyme, and assessing the viability of hypothetical pathways. Furthermore, insights for the next research objective involving application of computational chemistry to plant secondary metabolism are provided herein. This review will be helpful for plant scientists who are not well versed with computational chemistry.

## Introduction

Recent advances in life science have revealed many plant secondary biosynthetic pathways and have also contributed to establishing efficient microbial-based manufacturing systems that can potentially afford greater amounts of important plant secondary metabolites, including artemisinin (Ro et al., 2006; Paddon et al., 2013), opioids (Galanie et al., 2015), and cannabinoids (Luo et al., 2019), by introducing all of the biosynthetic genes into the host. These outstanding achievements are obviously based on long-standing biosynthetic studies. In opioid heterologous expression systems, not only enzymes from plants but also enzymes from other organisms were used, which are relevant to the expression levels and substrate specificity. Considering the progress of biosynthetic studies and synthetic biology, rational engineering of the existing biosynthetic pathways or designing the novel pathways to obtain desired products appears to be the next challenge. To achieve this objective, detailed mechanistic investigations of biosynthetic reactions are critical for the rational modification of biosynthetic pathways or enzymes.

Although various types of experimental methods, such as labeling experiments, X-ray crystallography, site-directed mutagenesis, omics studies (Rai et al., 2017, 2019), and genome editing, have been applied, clarifying entire biosynthetic pathways and mechanisms remains a challenge in plant science. Unlike microorganisms, biosynthetic genes do not form clusters in plants, which makes biosynthetic studies of plants difficult. Even finding a biosynthetic gene (enzyme) is a challenge. For example, thus far, in quinolizidine alkaloid biosynthesis, only two enzymes have been characterized for their skeletal formation, although studies on their biosynthesis have been carried out for over half a century (Bunsupa et al., 2012; Yang et al., 2017).

Recently, an increasing number of computational chemistry studies on natural product biosynthesis have been published, which provides new insights that cannot be discovered solely by experimental approach. However, among plant biologists and computational chemists, there is still a significant gap in understanding how computational chemistry is an effectual tool. Through several representative biosynthetic studies on terpene/terpenoid, alkaloid, flavonoid, and lignin, this review describes what can be clarified using computational chemistry and also describes the computational methods, which are widely used for the mechanistic investigation of biosynthetic reactions. An attempt to be comprehensive was made; however, apologies are offered in advance for any accidental oversights. Moreover, there are many computational predictions of NMR, UV, and CD spectra, and mechanistic investigations on fungal or bacterial secondary metabolism; however, these studies are not covered here since this review focuses on the “mechanistic investigations” of “plant” secondary metabolism.

## Theoretical Methods For Natural Product Biosynthesis Research

In plant science, cheminformatics such as KEGG (Kanehisa and Goto, 2000; Kanehisa et al., 2017, 2019) KNApSAcK (Afendi et al., 2011), etc., are widely utilized to predict biosynthetic pathways. However, computational chemistry is completely different from these database search approaches. In computational chemistry, the properties of compounds, such as free energy, spectrum, reactivity, and molecular orbitals, etc., are obtained by *ab initio* calculations based on the Schrödinger equation in molecular orbital (MO) calculations, the Kohn-Sham equation in density functional theory (DFT) calculations, and the Newton equation in molecular dynamics (MD) simulations (Jensen, 2017).

Although a variety of computational methods are available for the mechanistic investigation of chemical reactions, carefully choosing appropriate methods is important in terms of accuracy and computational expenses. Today, quantum mechanics (QM), i.e., MO or DFT, are mainly used for the evaluation of the reactivity or properties of small molecules. For mechanistic investigations using QM calculations, transition state (TS) search is initially performed, after which frequency calculation is performed to ensure that the TS has a single imaginary frequency. Finally, intrinsic reaction coordinate (IRC) calculation is carried out to obtain the reactant and product (Ishida et al., 1977; Fukui, 1981; Gonzalez and Schlegel, 1989; Page et al., 1990; Schlegel and Gonzalez, 1990). Today, hundreds of levels of theory are available. The choice of the level of theory is quite critical for computation accuracy in QM, which is dependent on the type of reactions or chemical structures. Many publications on benchmark tests exist, in which several combinations of density functional and basis set are tested against the one certain reaction or molecule to find the most accurate level of theory. For example, mPW1PW91/6-31+G (d,p)//B3LYP/6-31+G(d,p) has been used for the terpene-forming reaction based on the benchmark test reported by Matsuda et al. (2006). In this literature, many combinations of basis set and functionals were tested and compared with the experimental data. The results indicated that B3LYP/6-31+G(d,p) is the best for geometry optimization, whereas mPW1PW91/6-31+G(d,p) is the best for computing the free energy for the terpene-forming reaction.

In plant secondary metabolism, most of the biosynthetic reactions are thought to be catalyzed by enzymes; however, the system size, which QM calculations can treat, is usually up to a few hundred atoms. This means that biological macromolecules, i.e., enzymes, are too large to be calculated with QM. Theozyme calculation (Tantillo et al., 1998; Ujaque et al., 2002; Tantillo, 2010) is one way to estimate the enzymatic assistance toward the chemical conversion, in which the catalytic center, substrate, and several residues are picked up and subsequently subjected to DFT calculations (see Section “Theozyme Calculation Identified the Key Residue for Sesterfisherol Biosynthesis” for more detail). However, QM calculation is applied only for the isolated model, and the regions that could affect the chemical conversion are ignored. Thus, MD simulation is used for large macromolecule systems, which can simulate the time-dependent structure changes of enzymes; however, the free energy value is less accurate than that obtained using QM. For relatively high accuracy, quantum mechanics/molecular mechanics (QM/MM) is used for large macromolecule systems, in which the reaction system is divided into two regions: QM and MM. Generally, the catalytic center is calculated using QM, and the other parts of the enzyme are calculated using MM. Moreover, a state-of-the-art QM/MM MD is also utilized for mechanistic investigations.

## Terpenoids

Terpene/terpenoids are the largest natural product group. At least 80,000 terpene/terpenoids have been reported to date (Quin et al., 2014; Dickschat, 2016; Christianson, 2017). Most theoretical studies on terpene cyclization have been carried out by Tantillo and Hong. Although they have provided valuable insight into carbocation chemistry, only some of their works are presented in this review due to page limitations. One of the reasons for the considerable success of this compound group in computational chemistry is that terpenes consist of only carbons and hydrogens; therefore, strong interactions between the substrate and enzyme, such as hydrogen bonding, are not necessarily considered for its cyclization. In fact, the computed inherent reactivity shows good agreement with the experimental data for the terpene-forming reaction. Moreover, as was described above, a detailed benchmark test has been reported, which also supports the computation accuracy. In this section, several examples ranging from small systems to large systems will be discussed.

### Two Possible Cyclization Mechanisms Were Assessed Using Density Functional Theory Calculations

**Figure 1:**
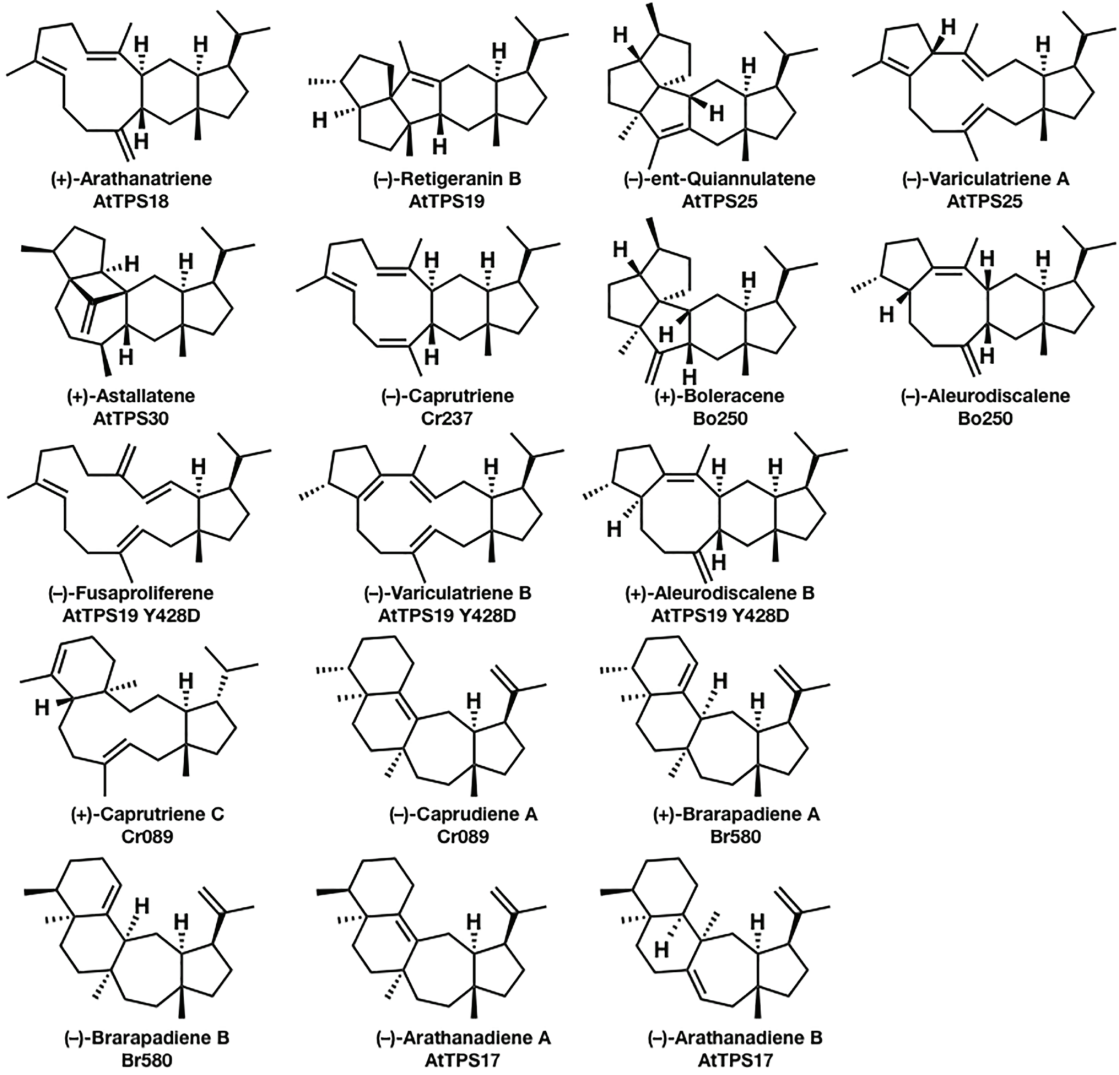
Seventeen sesterterpenes were isolated from plants based on the genome mining approach. The compounds’ names and their corresponding biosynthetic genes are shown below the structures. AtPTS is a terpene synthase from *Arabidopsis thaliana*; Cr is a terpene synthase from *Capsella rubella*; Bo is a terpene synthase from *Brassica oleracea*; and Br580 is a terpene synthase from *Brassica rapa*.

In comparison to mono-, sesqui-, di-, and triterpene, sesterterpenes are considered relatively minor terpenes, and only several biosynthetic enzymes have been characterized to date. In 2017, a genome mining approach to discovering the biosynthetic genes of sesterterpenes from *Arabidopsis thaliana, Capsella rubella, Brassica oleracea*, and *Brassica rapa* was reported by Huang et al. (2017), which strongly promoted the biosynthetic research of sesterterpenoids. They isolated (+)-arathanatriene, (−)-retigeranin B, (+)-astellatene, (−)-ent-quiannulatene, (−)-variculatriene A, (−)-caprutriene, (+)-boleracene, (−)-aleurodiscalene A, (+)-caprutriene C, (−)-caprudiene A, (+)-brarapadiene A, (−)-brarapadiene B, (−)-arathanadiene A, and (−)-arathanadiene B, and also characterized the corresponding biosynthetic enzymes (Figure 1). Interestingly, some of these compounds are originally found in microorganisms, suggesting the convergent evolution of biosynthetic genes.

**Figure 2:**
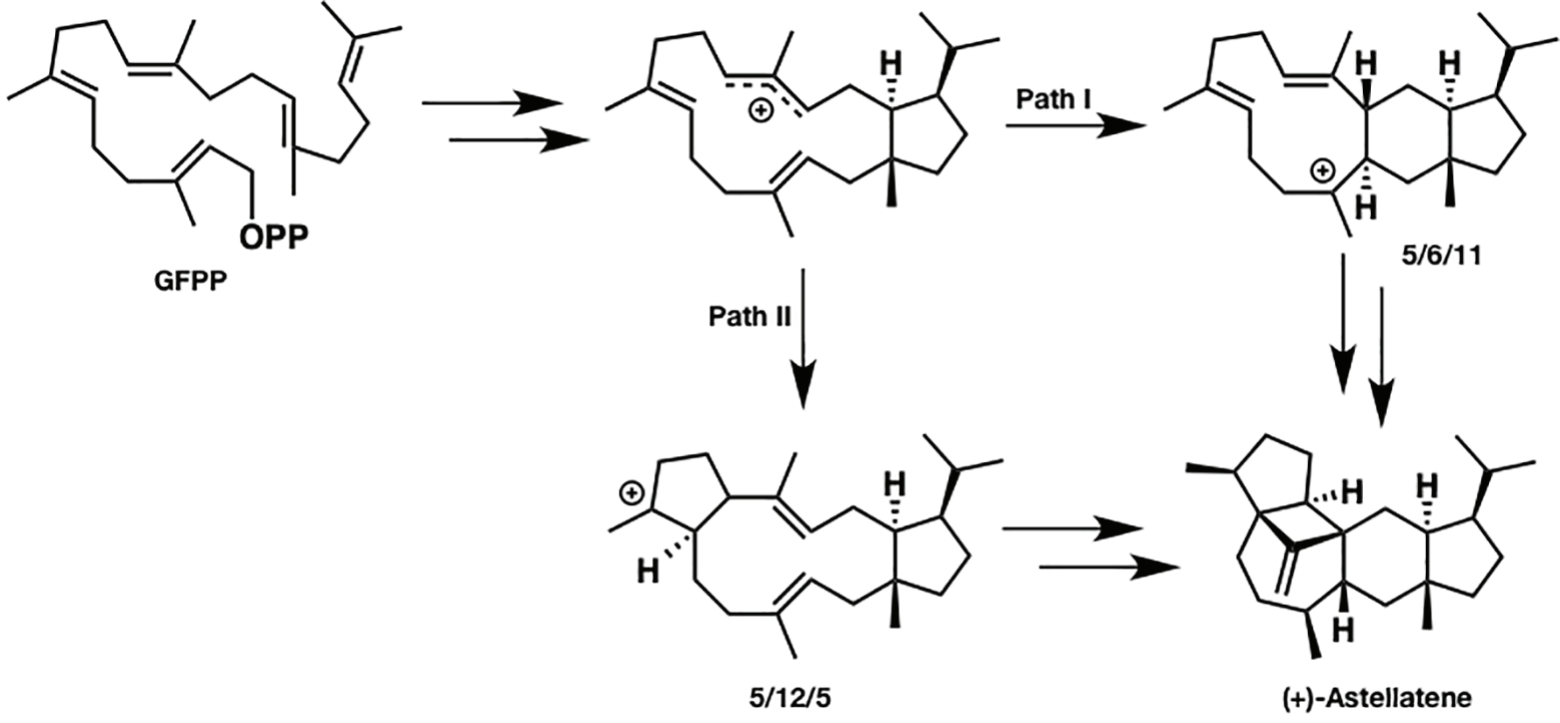
Two reasonable biosynthetic pathways of (+)-astellatene. In path I, 5/6/11 tricyclic skeletons are formed. Conversely, a 5/12/5 tricyclic skeleton is formed in path II.

Since sesterterpenes have more carbons than sesqui- and diterpenes do, their structure is more flexible and complicated, which makes the mechanistic investigation challenging. For example, two reasonable pathways can be proposed for (+)-astellatene formation (Figure 2). In path I, a 5/6/11 tricyclic skeleton is formed first, whereas path II involves the initial formation of a 5/12/5 tricyclic skeleton. Prior to this research, a labeling experiment was performed by Ye et al. in the biosynthesis of sesterfisherol, which is a similar sesterterpenoid. They could not experimentally distinguish between the 5/6/5 and 5/12/5 tricyclic skeleton formations because both paths are consistent with the labeling experiment results (Ye et al., 2015). Moreover, predicting a reasonable pathway based on presumable intermediates is difficult. For example, (+)-arathanatriene and (−)-caprutriene could be formed *via* path I, whereas (−)-variculatriene A and (−)-variculatriene B could be formed *via* path II, indicating that the experimental approach is not enough to answer this question. Accordingly, Huang et al. performed DFT calculations to assess the viability of the proposed biosynthetic pathways of (+)-astellatene and brarapadiene A (Huang et al., 2018).

Based on their computational results, both pathways are energetically viable; however, path I is inherently preferred since the highest energy barrier in path I is lower than that in path II by 6 kcal/mol. This study shows that DFT calculations can clarify the inherent reactivity of the carbocation intermediate in terpene biosynthesis, which could be useful for distinguishing the favored pathway. This combined experimental and computational approach is very useful, particularly when the reaction mechanism cannot be accessed solely using experiments.

### Density Functional Theory Calculation Revealed That the Initial Conformation Affects the Regio- and Stereoselectivity

In terpene cyclization cascade, the computed inherent reactivity is consistent with the experimental result, indicating that enzymes do not precisely manipulate the reactions step by step. However, conventionally, various kinds of terpenoids are synthesized from common isoprenoid substrates. Therefore, some may wonder how terpene cyclases can control the regio- and stereoselectivity without interacting with the intermediate during the cyclization.

To investigate the origin of the terpenoid structural diversity, Sato et al. carried out a detailed comparative study of two structurally similar sesterterpenes: sesterfisherol and quiannulatene, which were synthesized by NfSS and EvQS, respectively (Sato et al., 2018a). Based on their calculations, these sesterterpenoids are formed *via* the 5/12/5 tricyclic intermediate unlike for astellatene (Figure 2, path II). In addition, a careful comparison study clarified the conformational difference between the eight-membered rings in these two 5/12/5 intermediates. Here, we refer to the conformation of the quiannulatene’s 5/12/5 tricyclic intermediate (IM_Q) as conformation A, and that of sesterfisherol (IM_S) as conformation B.

**Figure 3:**
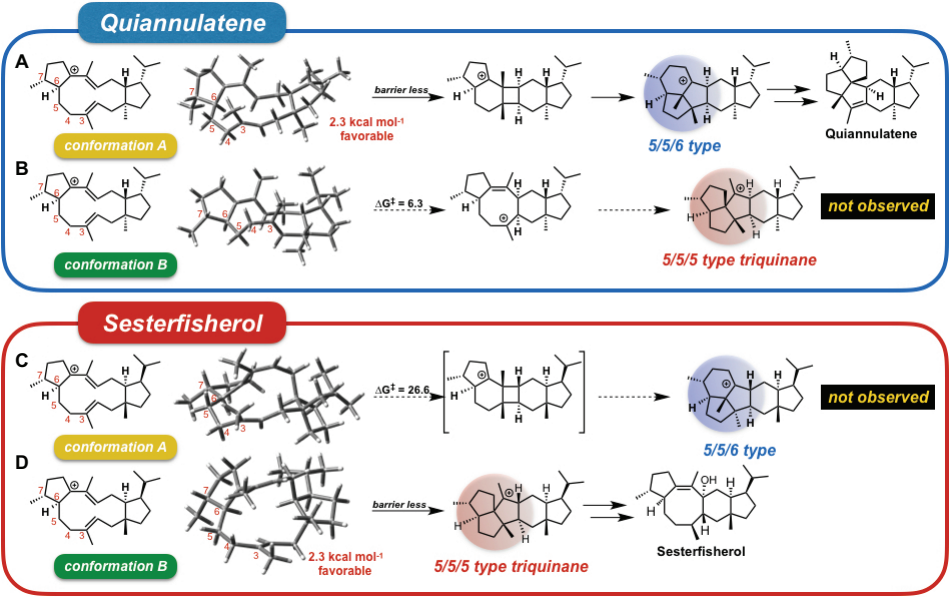
Conformational swapping changed the regioselectivity. This figure represents the regioselectivity of the 5/11/5 tricyclic intermediates in both quiannulatene and sesterfisherol biosyntheses, in each conformation. **(A)** Quiannulatene biosynthesis pathway in comformation A, **(B)** Quiannulatene biosynthesis pathway in comformation B, **(C)** Sesterfisherol biosynthesis pathway in conformation A, **(D)** Sesterfisherol biosynthesis pathway in conformation B.

They carried out “conformation swapping analysis” among these two sesterterpenoid intermediates (Figure 3). When IM_Q is in conformation B, 5/5/5-type triquinane formation proceeds, which was observed in sesterfisherol biosynthesis (Figure 3B). However, this triquinane formation reaction does not proceed inside EvQS, because conformation A is more stable than conformation B by 2.3 kcal/mol, and the activation energy of the 5/5/6-type condensation reaction is much lower. When IM_S is in conformation A, a 5/5/6-type condensation reaction proceeds, which is observed in quiannulatene biosynthesis (Figure 3C). However, this reaction cannot proceed inside NfSS because the energy barrier is too high (∆*G*^‡^ = 26.6 kcal/mol). This analysis suggests that the destination of the cyclization cascade is determined by the conformation of the intermediates. For each intermediate, the preferred conformation is different (A is preferred in quiannulatene biosynthesis, and B in sesterfisherol biosynthesis), which could be attributed to the stereochemistry of the 5/11 condensation positions (*trans*-fused in IM_Q while *cis*-fused in IM_S).

**Figure 4:**
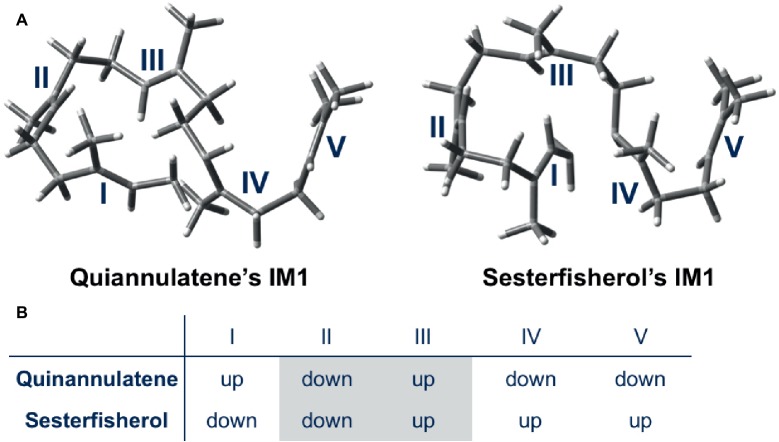
Initial conformations of GFPP in quiannulatene and sesterfisherol biosyntheses. **(A)** 3D representations of IM1 in the quiannulatene biosynthesis pathway and the sesterfisherol biosynthesis pathway. **(B)** The orientation of methyl groups attached to the double bonds I, II, III, IV, and V.

Moreover, a further detailed comparison was carried out for the whole cyclization cascade, from which the initial conformation was found to be critical for the selectivity (Figure 4). The orientation of the methyl group of each double bond in the initial conformation determines the stereochemistry of the intermediate; accordingly, the conformation of the intermediate is automatically fixed, and the destination of the cascade is automatically set. In conclusion, terpene synthase appears to regulate the regio- and stereoselectivity by fixing the initial conformation.

### Theozyme Calculation Identified the Key Residue for Sesterfisherol Biosynthesis

Generally, the main roles of terpene cyclases are (1) abstraction of pyrophosphate, (2) fixing the initial conformation (as was described above), (3) protecting the reactive intermediates from water, and (4) termination of the cyclization. The terpene-forming reaction is initiated by the elimination of the pyrophosphate group; afterward, the cyclization is driven by the inherent reactivity of the carbocation. However, the residue in the active site occasionally affects the carbocation intermediate in the cyclization cascade. Considering computational expenses, theozyme calculation is a great method for estimating the involvement of residues in the active site. Here, we show one example in which theozyme calculation and experimental validation worked successfully.

**Figure 5:**
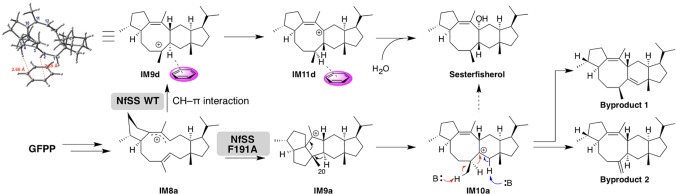
Summary of the biosynthetic pathways catalyzed by NfSS WT and NfSS F191A.

A detailed theoretical study of sesterfisherol biosynthesis was published by Sato et al. (Narita et al., 2017; Sato et al., 2018b). While they were exploring the cyclization mechanisms of sesterfisherol, a deep minima was found that required extraordinarily high energy, which was different from the activation energy in terpene biosynthesis. Subsequently, they examined the possibility of C–H π interactions (Hong and Tantillo, 2015), since terpene cyclase active sites are generally formed by aromatic and aliphatic residues. To estimate the C–H π interaction, a benzene ring was located around the carbocation center of the intermediate, and the transition state structure search was subsequently carried out. Consequently, an alternative pathway was found, in which the C–H π interaction lowered the activation energy and also avoided the deep minima (Figure 5).

**Figure 6:**
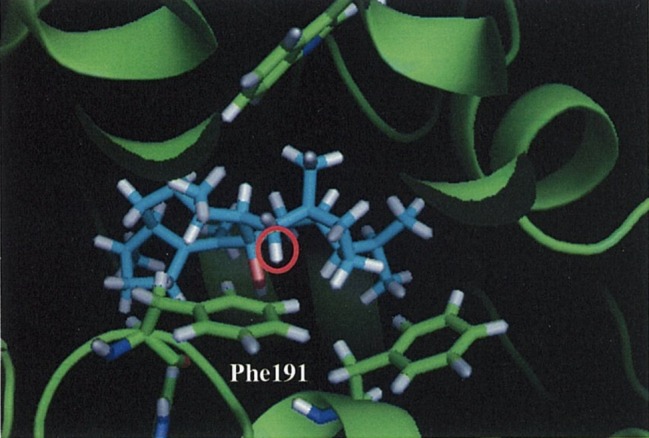
A carbocation intermediate and NfSS complex obtained by the docking simulation.

Notably, they also experimentally examined this C–H π interaction by site-directed mutagenesis. First, they constructed a homology model of NfSS and carried out the docking simulation. Afterward, several aromatic residue candidates that could form the C–H π interaction were found in NfSS’s active site (Figure 6). Due to their experiment, NfSS F191A produced new compounds instead of sesterfisherol (Figure 5). This result suggests that F191 is the key catalytic enzyme in sesterfisherol biosynthesis.

Interestingly, two of the products produced by NfSS F191A are thought to be derivatives of computationally predicted intermediates (Byproduct 1 and Byproduct 2), which could be deprotonated by the pyrophosphate adjacent to the active site.

As we have shown in this section, the combination of the theozyme calculation, homology modeling, docking simulation, and site-directed mutagenesis can be a powerful tool for finding the key catalytic residues. We think this approach can accelerate the mechanistic studies of biosynthetic enzymes in plant secondary metabolism.

### High-Resolution All-Atom Model of Terpene Synthase

As was described above, terpene synthases play an important role in pre-organizing the initial conformation of the substrate. However, building molecular models of entire terpene-forming reactions within an active site remains a challenge. Major et al. mentioned the following in their review, “A crucial question in any study of terpene synthases is that of the correct binding mode. Indeed, crystal structures of terpene synthases often contain substrates bound in unreactive conformations, partly due to the stickiness of the hydrocarbon moiety of the substrate and its lack of hydrogen bond potential. Thus, there is often great uncertainty regarding the correct binding mode when commencing multi-scale simulation projects of terpene cyclases” (Major et al., 2014). To tackle this problem, O’Brien et al. combined QM calculation and computational docking with Rosetta molecular modeling suite and reported the high-resolution all-atom models of epi-aristolochene synthase (TEAS) from *Tobacco* (O’Brien et al., 2016) and (+)-bornyl diphosphate synthase from *Salvia officinalis* (O’Brien et al., 2018).

**Figure 7:**
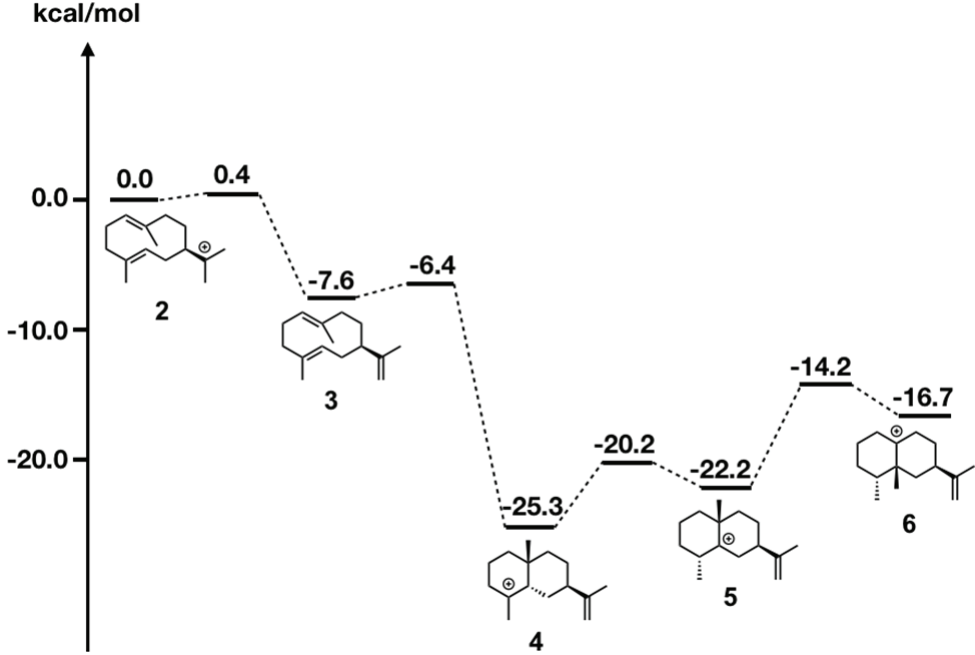
Energy diagram of epi-aristolochene biosynthetic pathway. Relative energies to intermediate 2 are shown in kcal/mol.

Due to page limitations, we briefly explain their methodologies here (please refer to the original paper for more details). In the study, they initially carried out DFT calculations along with the generally accepted biosynthetic pathway of epi-aristolochene formation (Figure 7). All computed carbocation intermediates were subsequently subjected to conformational search using molecular mechanics force field (MMFF). These conformation libraries, containing over a hundred conformers for each carbocation intermediate, were subsequently optimized using DFT calculations, and low energy structures within 5 kcal/mol were used for the docking simulation. For the preparation of the protein structure, the X-ray crystal structure was minimized using a constrained FastRelax (Conway et al., 2014) procedure from the Rosetta modeling suite (Meiler and Baker, 2006; Richter et al., 2011). The diphosphate/magnesium complex extracted from the TEAS crystal structure was docked along with previously generated conformer libraries into the relaxed crystal structure using the chemically meaningful constraints. To ensure that the sampling was sufficient, 2,500 docking runs per catalytic orientation (motif) were performed. The resulting structures were combined and subsequently subjected to filtering based on three explicit constraints, involving (1) the departing diphosphate oxygen that results in the carbocation; (2) deprotonation of carbocation intermediate 2; and (3) protonation of carbocation intermediate 3.

**Figure 8:**
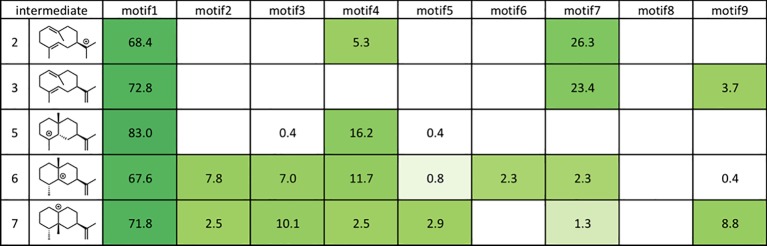
Docking results using Rosetta. The structures of the intermediates are shown on the left. The darker the green in each box, the higher the percentage of low energy structures that are found in that catalytic orientation. If no low energy solutions were found for a particular intermediate, then no value was given. The number is the percentage of total low energy structures found for the catalytic motif when docking a particular intermediate.

As shown in Figure 8, motif 1 is abundant in all intermediates, suggesting that this orientation is the most reasonable docking mode in TEAS. Furthermore, they carried out the RMSD calculation on the motif 1 structures in all intermediates and revealed the least movable docking mode during the cyclization reaction. Interestingly, only a few orientations are enriched in the early stage of this biosynthesis, whereas several motifs are enriched in the late stage of this enzyme reaction. In addition, the RMSD value was increased in the late stage of this cyclization reaction. These are consistent with the generally accepted concept that the substrate affinity decreases as the reaction proceeds. We think this method is applicable for future efforts to carry out the rational redesign of reaction specificity of this class of enzymes.

### Quantum Mechanics/Molecular Mechanics Molecular Dynamics Calculation Revealed the Substrate Specificity of Geranyl Diphosphate Synthase

Although the inherent reactivities of the carbocation intermediates, computed by DFT calculations, are consistent with the experimental results, terpene cyclases occasionally affect the carbocation intermediates, as shown in Section “Theozyme Calculation Identified the Key Residue for Sesterfisherol Biosynthesis”. Therefore, the calculation including both the substrate and whole protein structure might be required to estimate the significance of the enzymatic support. For this purpose, the QM/MM method is widely used due to its computational cost. Here, we introduce a detailed study of geranyl diphosphate synthase (GPPS) from *Mentha piperita*, reported by Wu and Xu (Liu et al., 2014).

GPPS accepts isopentenyl diphosphate (IPP) and dimethylallyl diphosphate (DPP) as substrates and yields geranyl diphosphate (GPP), which is the first step of the chain elongation in isoprenoid biosynthesis. In the current study, they carried out MD simulations to reveal the mechanism of an “open-closed” conformation change of the catalytic pocket in the GPPS active site and identified a critical salt bridge between **Asp91** (in loop 1) and **Lys239** (in loop 2), which is responsible for opening or closing the catalytic pocket. In addition, the small subunit regulates the size and shape of the hydrophobic pocket to flexibly host substrates with different shapes and sizes (DPP/GPP/FPP, C_5_/C_10_/C_15_). Furthermore, QM/MM MD simulations were carried out to explore the binding modes for the different substrates catalyzed by GPPS. GPPS is known to be a bifunctional enzyme and can catalyze GPP, GGPP, and a negligible amount of FPP formation. QM/MM MD simulation revealed that the distances and angles between two substrates are critical for the reaction. These parameters are similar when GPP or GGPP is produced, whereas the reverse is the case when FPP is produced. This study shows how QM/MM MD simulation is effective for clarifying the enzymatic effect toward the reaction in plant metabolism. Moreover, the key residues Asp91, Lys239, and Gln156, which could be good candidates for the site-directed mutagenesis, were found based on the computation.

As was described in Section “High-Resolution All-Atom Model of Terpene Synthase,” building the high-resolution all-atom model of terpene synthase has been challenging for a long time, which is one reason there are only a few examples of QM/MM calculations of terpene synthase. We expect an increased number of reports on the all-atom modeling, using QM and Rosetta modeling suite, in the near future, which would promote QM/MM calculations. Moreover, we hope this kind of research will facilitate the mechanistic investigation and rational engineering of the biosynthetic pathways in plants.

## Alkaloids

Unlike terpenoids, alkaloids have polar moieties that can form strong interactions with the enzyme. Therefore, more careful model construction is necessary, such as theozyme, MD simulation, or QM/MM. Accordingly, few theoretical studies on alkaloid biosynthesis have been reported to date. Here, we introduce four theoretical studies on the inherent reactivity in alkaloid biosynthesis.

### Density Functional Theory Calculation Revealed a Favorable Mechanism for β-Carboline Formation Catalyzed by Strictosidine Synthase

**Figure 9:**
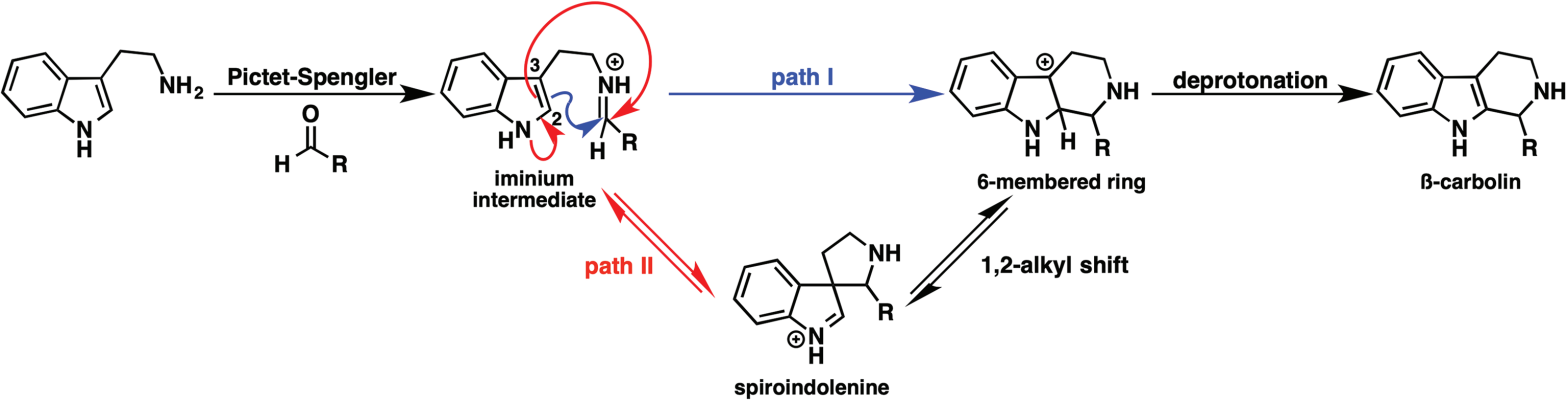
Possible pathways for β-carboline formation.

Strictosidine is an important common intermediate that can be converted to many kinds of plant indole monoterpenoid alkaloids, such as ajmalicine, quinine, vinblastine, reserpine, camptothecin, and vincamine. The Pictet-Spengler condensation reaction of tryptamine with secologanin, catalyzed by strictosidine synthase, has been intensively studied, and several X-ray crystal structures have been reported. The Pictet-Spengler reaction consists essentially of two steps. First, an electron-rich aromatic amine attacks the aldehyde of secologanin to form an iminium intermediate. Second, an aryl amine attacks the electrophilic iminium to yield a positively charged intermediate that is then deprotonated to yield a β-carboline product. Although the ligand-bound crystal structure is available, the detailed mechanism of β-carboline formation remains unclear. Notably, carbon 2 and 3 of the indole moiety are nucleophilic; therefore, two possible mechanisms can be written (Figure 9). In path I, carbon 2 attacks the iminium moiety, after which the direct six-membered ring formation proceeds. In path II, carbon 2 attacks the iminium moiety, thereby forming spiroindolenine, which is subsequently converted to β-carboline by a 1,2-alkyl shift. To clarify which pathway is more reasonable, Maresh et al. carried out DFT calculations (Maresh et al., 2008).

**Figure 10:**
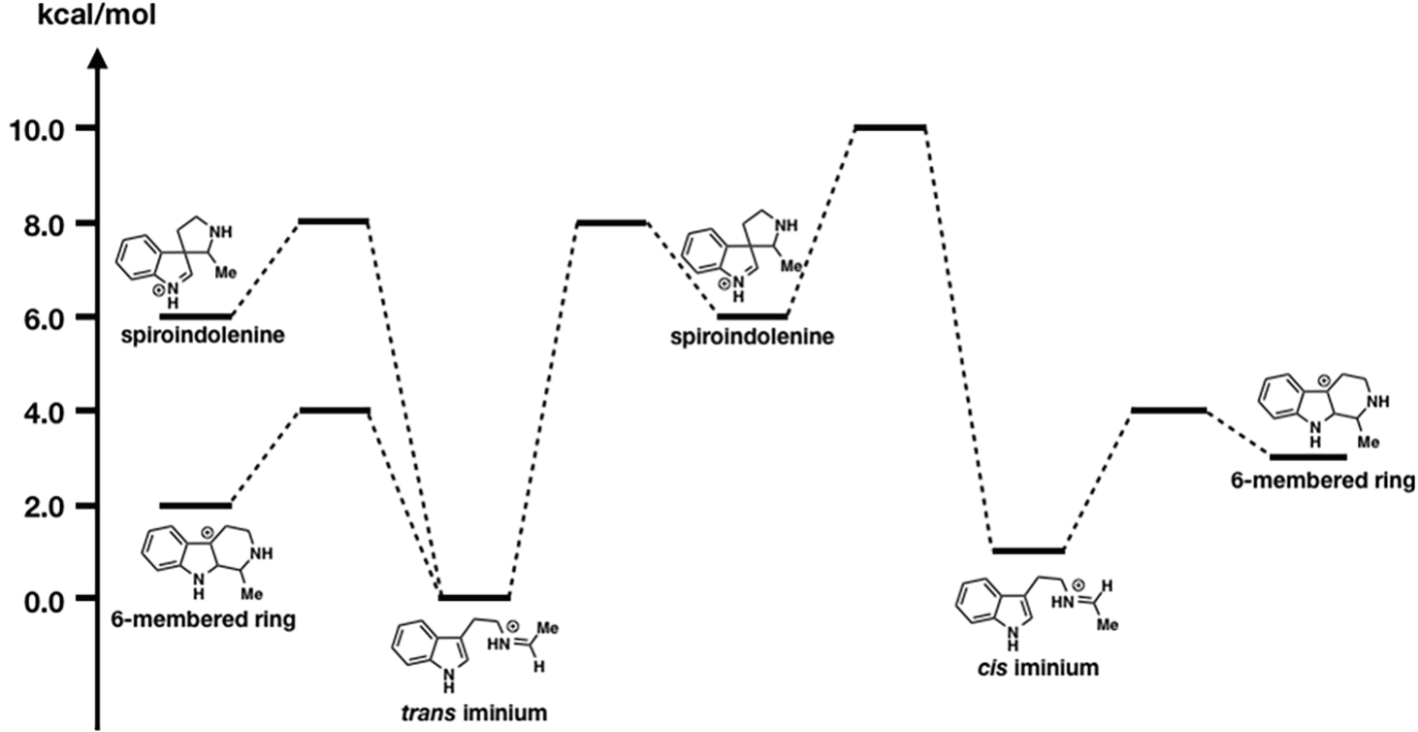
The energy diagrams of the six-membered ring formation from spiroindolenine. Relative free energies to trans iminium are shown in kcal/mol.

As shown in Figure 10, the formation of the six-membered ring is several orders of magnitude faster than spiroindolenine formation, which is consistent with the empirical rule: that 6-endo-ring closures are favored over 5-endo-trig cyclization. However, the formation of the spiroindolenine intermediate has been observed by isotopic scrambling. In addition, spiroindolenine formation requires only 8 kcal/mol, and it appears to occur under ambient conditions. Therefore, if the deprotonation is slow enough, spiroindolenine can be formed during the course of the reaction. Moreover, this calculation also suggests that the 1,2-alkyl shift that connects iminium spiroindolenine and the six-membered ring intermediate requires significantly high energy, which does not contribute to the mechanism.

Even though we obtained X-ray crystal structures, revealing the detailed reaction mechanisms is often challenging. This study indicates that the combined strategy of computations and kinetic isotope experiments is quite effective in distinguishing two possible pathways. We hope this approach will be widely utilized in the study of plant secondary metabolism.

### Camptothecin E Ring Opening Reaction

Camptothecin (CPT) is a plant alkaloid that was originally isolated from the Chinese tree, *Camptotheca acuminate*, in 1966 (Wall et al., 1966). It features a planar pentacyclic ring structure with a pyrrolo [3-4-β] quinolone moiety (rings A, B, and C), a conjugated pyridine group (ring D), and one α-hydroxy lactone ring (ring E); consequently, it has received interest from scientists due to its remarkable anticancer activity in preliminary clinical trials. Although two CPT analogs, topotecan and irinotecan, have been approved and are currently used in cancer chemotherapy, the pharmacological investigation into CPT had been suspended on one occasion due to the poor solubility of CPT in water and most organic solvents (Gupta et al., 1995; Wu and Liu, 1997; Wall, 1998).

The stabilities of CPT and its derivatives in solution were reported to be highly pH-dependent by several experimental analyses. In addition, CPT and its derivatives tend to aggregate in solution, particularly in dimer formation. One of the key factors is the E-ring-opening reaction that can proceed at neutral pH. Zou et al. carried out DFT calculations to obtain the theoretical basis for this E ring opening reaction (Zou et al., 2013). In their study, they revealed (1) that the E-ring opening reaction can proceed under the physiological pH (∆G^‡^ = 12.94 in aqueous), (2) the solvation effect, and (3) the substitution effect. This study is a good example of how computational chemistry is an effective tool for revealing the degradation process of plant alkaloids and for examining their instability.

### Biosynthetic Dipolar Cycloaddition: Daphniphyllum Alkaloids, Flueggine A, and Virosaines

Cycloaddition is one of the most important reactions for skeletal formation in both total synthesis and biosynthesis of natural products. There have been many theoretical studies on Diels-Alderase which provide insightful mechanistic approaches that cannot be easily addressed by experimental approaches. These reports also show that the inherent reactivity of a molecule is relevant to many cyclization reactions promoted by enzymes. Here, we show two theoretical studies of plant alkaloids from *Daphniphyllum* (Tantillo, 2016) and *Flueggea* (Painter et al., 2013).

**Figure 11:**
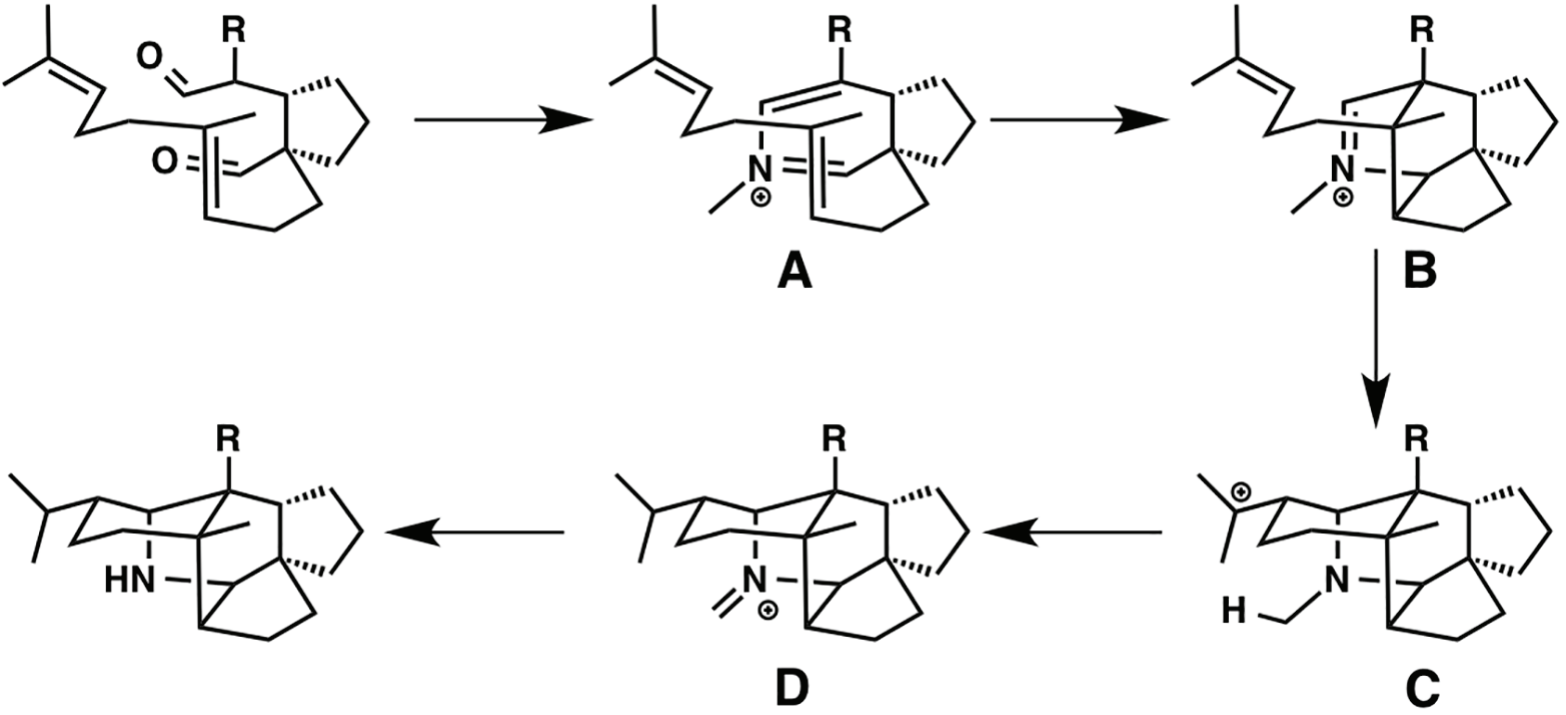
Biosynthetic pathway of *Daphniphyllum* alkaloids.

The development of cascade polycyclizations by Heathcock and co-workers, to construct *Daphniphyllum* alkaloids, is a milestone in biomimetic total synthesis, in which bicyclic intermediate A is thought to be converted into tetracyclic intermediate D *via* intermediates B and C (Heathcock et al., 1992; Heathcock, 1996). Based on their calculations, A is converted to B with an activation energy of only 7.1 kcal/mol *via* concerted [4 + 2] cycloaddition (aza-Diels-Alder reaction). This result represents a potential biological Diels-Alder reaction for which enzymatic barrier lowering would not be required. It also demonstrates that enzymatic preorganization of the substrate is not required for a successful reaction, presumably because most conformers are unreactive. Interestingly, B is directly converted to D *via* ene reaction, in which C is not a minima but a transient structure (Figure 11).

**Figure 12:**
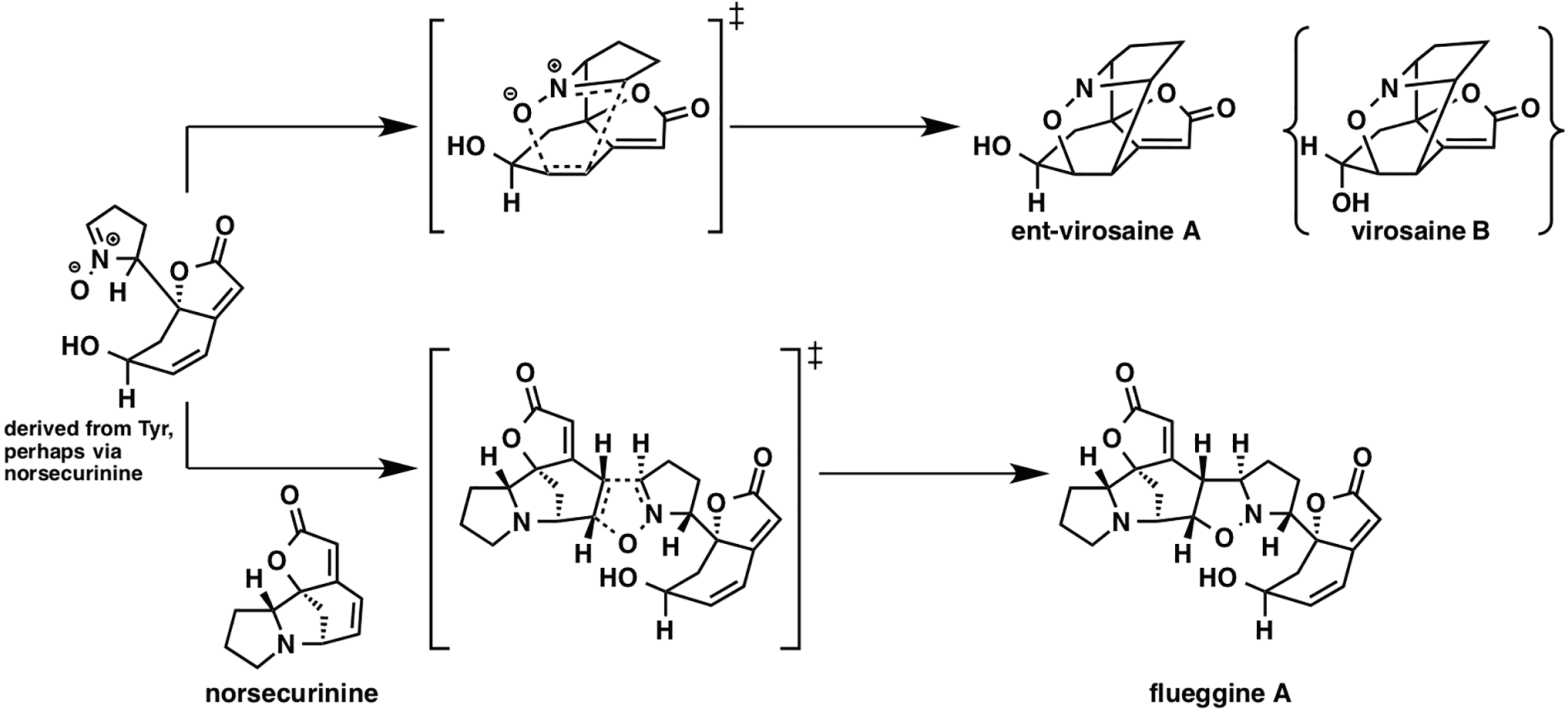
Plausible biosynthetic pathways of virosaine A, B, and flueggine A.

While there have been many studies on [4 + 2] cycloaddition reactions, other types of cycloadditions received less attention. Flueggine A and virosaine alkaloids, isolated from *Flueggea virosa*, are thought to be synthesized from a common precursor that could be derived from norsecurinine or tyrosine (Figure 12). Interestingly, this proposed biosynthetic pathway involves a nitrone-alkene (3 + 2) cycloaddition reaction.

In the referenced study, they examined all possible eight stereoisomers for flueggine A formation. The predicted energy barrier for the cycloaddition in water *via* this transition state structure is 10.3 kcal/mol, which is lowest among all the transition state structures for this reaction, implying that selectivity control by the enzyme is not required. Based on the distortion interaction model, this transition state has both considerably small distortion energy and considerably large interaction energy. They also examined virosaine formation, and the activation energy was only ~1 kcal/mol, indicating that enzymatic assistance is unnecessary.

It is always difficult to ascertain if the Diels-Alder reaction involved in the proposed biosynthetic pathway is enzymatic or not. However, computational chemistry provides us with the energy landscape of the reaction, which can assist in narrowing down the candidate biosynthetic genes in plant science.

### Elucidation of ∆^1^-Piperideine Dimer Formation in Quinolizidine Alkaloid Biosynthesis

**Figure 13:**
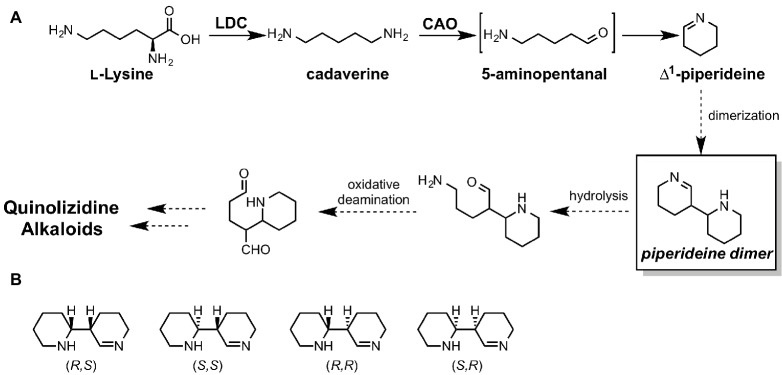
**(A)** proposed biosynthetic pathways of quinolizidine alkaloids. LDC stands for lysine decarboxylase, and CAO stands for copper amine oxidase. **(B)** Four possible stereoisomers of piperideine dimers.

Unlike for fungi and bacteria, revealing whole biosynthetic genes relevant to a plant secondary metabolite is still quite challenging. Quinolizidine alkaloids (QAs), a subclass of Lys-derived plant alkaloids widely distributed in *Leguminosae*, have been intensively studied for over half a century. However, most of their biosynthetic genes, enzymes, and intermediates remain unknown. Particularly, in the quinolizidine skeletal formation process, which is thought to be common in all QA biosynthesis, only two enzymes, L-lysine decarboxylase (LDC) (Bunsupa et al., 2012) and copper amine oxidase (CAO) (Yang et al., 2017), have been identified to date (Figure 13A). Therefore, there is no valid information on how many genes, enzymes, and reactions are required for production of QAs after ∆^1^-piperideine formation. In a plausible biosynthetic pathway of QAs, 5-aminopentanal is spontaneously converted to ∆^1^-piperideine (Figure 13A). The dimerization of ∆^1^-piperideine has been proposed; however, it is unclear whether or not it requires enzymatic assistance. Therefore, Sato et al. carried out theoretical investigations to uncover the biosynthetic mechanism of ∆^1^-piperideine dimerization (Sato et al., 2018c). There are four possible stereoisomers for the piperideine dimer, i.e., *(R,R), (S,S), (R,S)*, and *(S,R)* (Figure 13B). In the mentioned study, they constructed a model that has two molecules of ∆^1^-piperideine and two to four molecules of water for each isomer.

The results of the DFT calculations indicate that piperideine dimerization spontaneously proceeds under neutral conditions and yields only (R,R) or (S,S) dimers. (R,S) and (S,R) dimer formations require considerably higher energy barriers; therefore, they cannot be formed under neutral conditions. Based on the previous literature, tetrahydroanabasin, which could be formed from the (R,R) piperideine dimer through isomerization, was isolated from a QA producing plant. Therefore, (R,R) piperideine formation could be favored in a QA producing plant. This stereochemistry is also consistent with the other QA derivatives. For example, (−)-lupinine and (+)-epilupinine could be synthesized from (R,R)-piperideine and (R,S)-piperideine dimers, respectively. However, we cannot rule out the possibility that the enzyme assists this dimerization reaction since the (S,S)-piperideine dimer has not yet been isolated. Moreover, nature occasionally uses an enzyme for the reaction that can spontaneously proceed under aqueous conditions (Chen et al., 2018).

In the study of QA biosynthesis, differential expression analysis was performed to search for LDC genes, which also provided dozens of other candidate genes. The combination of computational chemistry and expression-based approach could be a significantly powerful tool for narrowing down the candidate genes.

## Other Secondary Metabolites

Unlike the case with other natural product groups, there are only a few mechanistic investigations on flavonoid and lignin biosynthesis, although there are many studies on spectrum prediction regarding flavonoid. Here, we introduce one study about anthocyanin biosynthesis and three studies about lignin biosynthesis.

### A Long-Standing Issue in Anthocyanin Biosynthesis Was Solved Using Density Functional Theory Calculations

**Figure 14:**
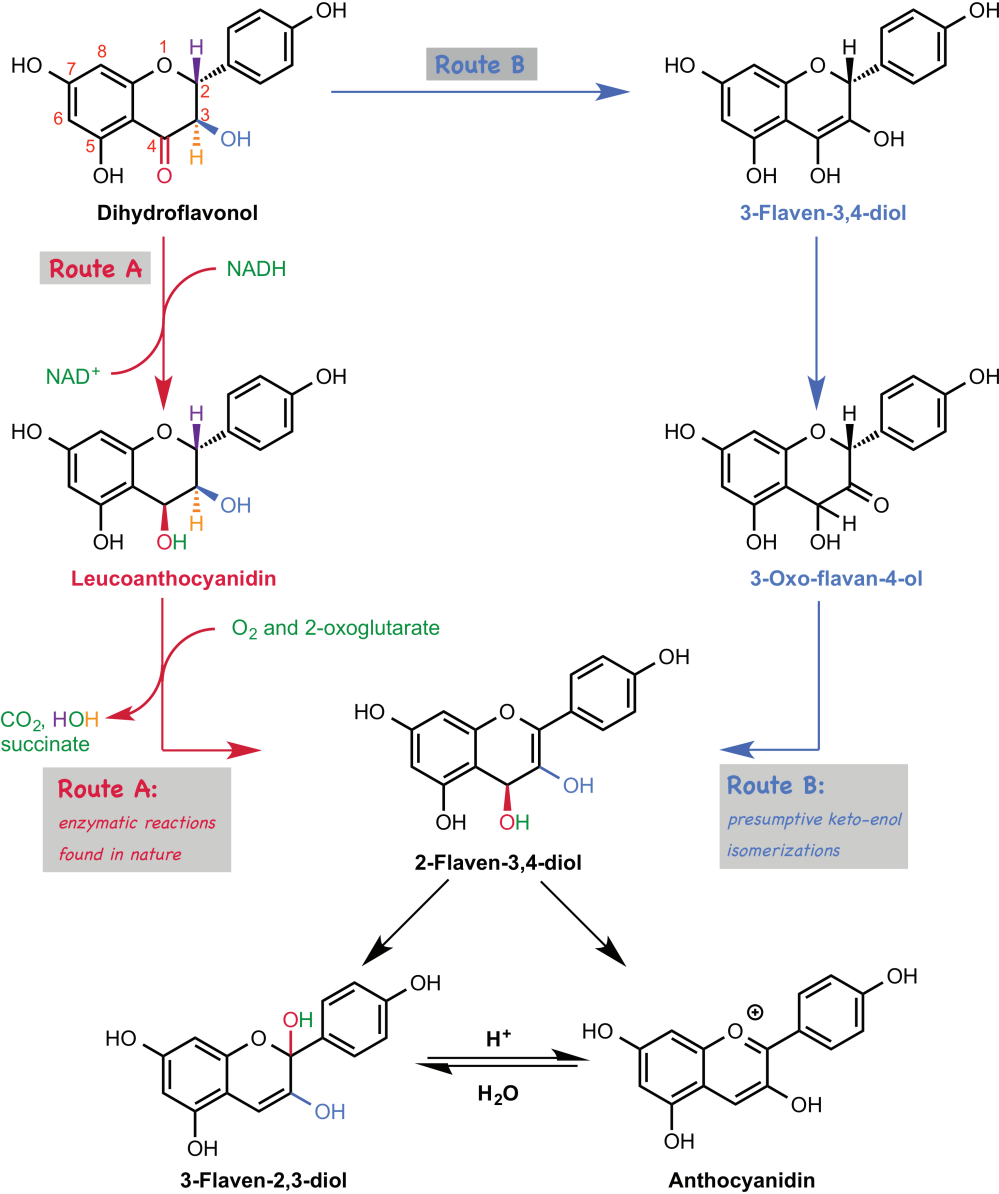
Two possible pathways for anthocyanidin biosynthesis. In route A, the conversion of dihydroflavonol to anthocyanidin is catalyzed by two enzymes, such as dihydroflavonol reductase (DFR) and anthocyanidin synthase (ANS). In route B, dihydroflavonol is converted to anthocyanidin by a three-step tautomerization reaction.

The biosynthetic pathways of flavonoids have been intensively studied, and related genes, enzymes, and intermediates have been characterized. In the late stage of the biosynthesis of anthocyanin, dihydroflavonoid is converted to anthocyanidin, which is catalyzed by two enzymes, dihydroflavonol reductase (DFR) (Gong et al., 1997) and anthocyanidin synthase (ANS) (Figure 14, Route A; Saito et al., 1999; Nakajima et al., 2001; Turnbull et al., 2004). Interestingly, reduction proceeds just after the oxidation in this conversion, which appears to be a detour pathway and energy consuming, because the oxidation state does not change after these two reactions. An alternative non-enzymatic pathway, that is a simple spontaneous tautomerization, was proposed (Figure 14, route B). However, this spontaneous pathway does not proceed inside the plant because the suppression of genes leads to a decrease in the amount of anthocyanidin. Therefore, the question about this biosynthetic pathway is why this tautomerization pathway is not feasible. To answer this question, Sato et al. carried out DFT calculations (Sato et al., 2018d).

Based on their calculations, the first tautomerization *via* the non-enzymatic pathway requires ca. 30 kcal/mol, which is too high to be achieved under ambient conditions. This is due to the instability of the transition state structure that has a highly electron rich en-diol moiety adjacent to the electron-rich aromatic ring. Interestingly, dihydroflavonol without hydroxyl groups at the 5′ and 7′ positions require lower activation energies than the original one does, indicating that the hydroxyl group is the natural controller of the yield of anthocyanidin. Moreover, the first tautomerization requires less activation energy under the acidic conditions, suggesting that this tautomerization requires enzymatic support. They searched for enzymes that could catalyze this type of tautomerization using the KEGG database, but no enzyme was found.

Furthermore, they investigated the later part of the anthocyanin biosynthetic pathway. However, interestingly, their DFT calculation suggests that 2-flaven-3,4-diol is directly converted to anthocyanidin under acidic conditions, which was originally thought to be converted *via* 3-flaven-2,3-diol formation (Figure 14). This new finding is consistent with the experimental result in the previous literature, in which hydrochloride was used to terminate the ANS reaction, and 3-flaven-2,3-diol was not detected.

As we described here, computational chemistry can simulate a reaction that cannot actually occur and can also easily delete the functional groups to assess their effect on the reaction, which is quite difficult in organic synthesis. This study emphasizes the usefulness of the computational approach to test the hypothetical pathways.

### Lignin

Unlike the other types of natural products that are mentioned above, only a few examples were reported for the mechanistic investigation of lignin biosynthesis. The theoretical investigation of lignin biosynthesis is challenging for some reasons, i.e., radical reaction, branched polymer structure, various kinds of coupling products, etc. To reveal the mechanism of lignin biosynthesis, a consecutive theoretical study has been reported by Sangha et al. (2012, 2014, 2016).

The initial step of lignin biosynthesis is initiated by the radical coupling reaction of monolignols. Besides the reactive oxygen, the monolignol derived radicals are reactive at the C1, C3, C5, and β positions due to the delocalization *via* the conjugated π system. Conventionally, lignin polymerization, catalyzed by peroxidases and laccases, takes place in three steps: (1) monolignol binding to the enzyme active site, (2) H_2_O_2_-mediated oxidation at the active site to form radicals, and (3) radical coupling reaction to form lignin polymers.

**Figure 15:**
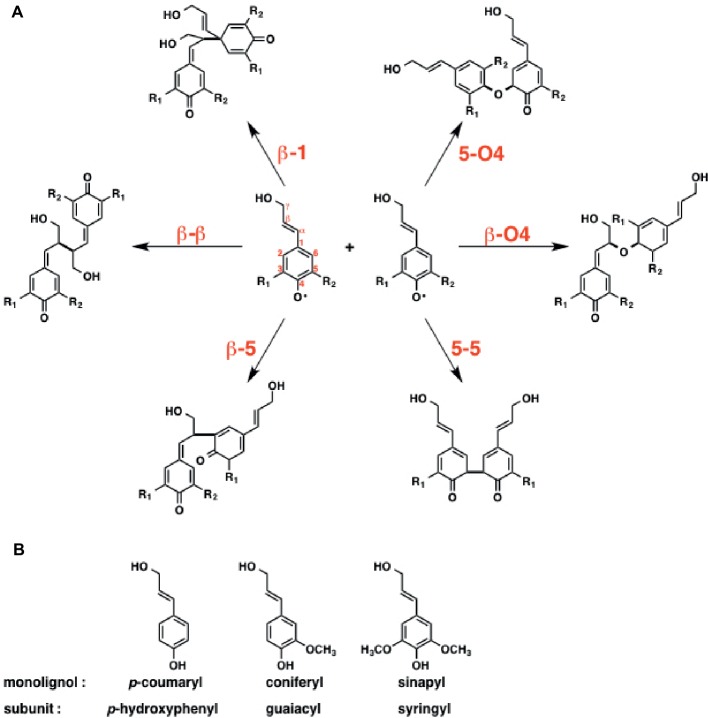
Initial step of radical-radical coupling of monolignol radicals during lignin biosynthesis. **(A)** Possible coupling mechanisms in lignin biosynthesis. **(B)** Three main monolignols that constitute lignin structure.

To reveal the inherent reactivity of monolignol, Sangha et al. initially carried out DFT calculation for the six possible radical coupling products, as shown in Figure 15A. The result indicates that the formation of β-O4, β-β, and β-5 type monolignol radical couplings is enthalpically favored over those of others, which is consistent with the experimental data suggesting that these three bonds are most common in natural lignin.

Next, they focused on horseradish peroxidase C (HRPC), which triggers the radical polymerization cascade in lignin biosynthesis. Lignin’s subunits can be classified into three groups; guaiacyl (G), syringyl (S), and *p*-hydroxyphenyl (H) (Figure 15B). The ratio of the subunits is relevant to the efficiency of the deconstruction of biomass. Therefore, they examined the binding affinity of three monolignols, including *p*-coumaryl, coniferyl, and sinapyl, toward the horseradish peroxidase C (HRPC). To answer this question, they carried out MD simulations. The results indicated that the binding affinity of the monolignols toward HRPC decreases in the order of *p*-coumaryl > sinapyl > coniferyl alcohol.

Since lignin biosynthesis is a combination of simple radical coupling reactions, many possible reaction pathways can be proposed. As we have shown in this section, computational chemistry can reveal the inherent reactivity and can provide a rational explanation for substrate specificity, which is helpful for narrowing down the candidate structures. We believe that computational chemistry can eliminate the hindrances to precise mechanistic investigations of radical coupling reactions in lignin biosynthesis.

## Summary and Perspectives

As we have shown in this review, computational chemistry can be a powerful tool for revealing the biosynthesis of secondary metabolites in plants. Unlike cheminformatics, computational chemistry provides not only reasonable reaction pathways and energy barriers but also many new insights, as described above. For example, ring cyclization order in astellatene biosynthesis, key catalytic residues of biosynthetic enzymes, the controlling mechanism of regio- and stereoselectivity in quiannulatene biosynthesis, docking mode in the active site of terpene synthase, detailed reaction mechanisms of piperideine dimerization, etc., which cannot be achieved solely by traditional experimental methods.

Although computational approaches using QM calculation, MD simulation, and QM/MM are well established in terpene-forming reactions and cycloadditions, only a few preliminary studies on other types of plant secondary metabolism have been reported until now. Many studies on other types of natural products are required to sophisticate this powerful computational approach. Particularly, more reports on the oxidation reactions, mainly catalyzed by P450, FMO, or iron-dependent enzymes (Nakashima et al., 2018), are desired because oxidation is one of the key reactions facilitating the structural diversity and complexity of plant secondary metabolites.

As was mentioned in Section “Introduction,” the next goal of these applicable studies using computational chemistry in natural product biosynthesis is to engineer or design novel biosynthetic pathways and enzymes to obtain desired products. One approach to achieve this objective is swapping the biosynthetic enzymes (genes) with other genes that can accept the biosynthetic intermediate, which could produce novel natural products. Another approach could be to design novel enzymes by computational chemistry (Kiss et al., 2013). The methodology of *de novo* enzyme design was reported by a decade ago, although it has not yet been applied to plant secondary metabolism engineering. They published three remarkable examples of artificially designed enzymes: retro-aldolase (Jiang et al., 2008), Kemp elimination enzyme (Röthlisberger et al., 2008), and Diels-Alderase (Siegel et al., 2010). In their method, they initially designed the catalytic cycle. Subsequently, they carried out DFT (theozyme) calculations to obtain the transition state structures. The next step involved searching the template protein, which has enough space in its cavity to accommodate the transition state structure. Finally, the enzyme was designed using Rosetta, which is also used in the terpene docking simulation, as described above. They successfully designed a desired artificial enzyme that was nonexistent. Moreover, they successfully improved the catalytic ability of those enzymes using directed evolution for several generations. We believe this kind of approach is very useful for the efficient production of novel bioactive plant secondary metabolites. We think terpene synthase is a good candidate for designing the artificial enzyme, since (1) the computational approach is well developed, (2) many transition-state structures are already available, (3) substrate is commercially available, and (4) most of the terpenes have unique biological activities. We hope that more biosynthetic studies utilize a computational chemistry-based approach for mechanistic investigations.

## Author Contributions

HS wrote entire manuscript under the supervision of KS and MY.

### Conflict of Interest Statement

The authors declare that the research was conducted in the absence of any commercial or financial relationships that could be construed as a potential conflict of interest.
